# Impact of Extracellular Matrix-Related Genes on the Tumor Microenvironment and Prognostic Indicators in Esophageal Cancer: A Comprehensive Analytical Study

**DOI:** 10.1155/2024/3577395

**Published:** 2024-07-25

**Authors:** Yinghong Wu, Wenjie Hu, Zhihong Jia, Qiying Zhu, Jinghui Xu, Liang Peng, Renjie Wang

**Affiliations:** The Second People's Hospital of Jingdezhen, Jingdezhen 333000, Jiangxi, China

## Abstract

Esophageal cancer is a major global health challenge with a poor prognosis. Recent studies underscore the extracellular matrix (ECM) role in cancer progression, but the full impact of ECM-related genes on patient outcomes remains unclear. Our study utilized next-generation sequencing and clinical data from esophageal cancer patients provided by The Cancer Genome Atlas, employing the R package in RStudio for computational analysis. This analysis identified significant associations between patient survival and various ECM-related genes, including IBSP, LINGO4, COL26A1, MMP12, KLK4, RTBDN, TENM1, GDF15, and RUNX1. Consequently, we developed a prognostic model to predict patient outcomes, which demonstrated clear survival differences between high-risk and low-risk patient groups. Our comprehensive review encompassed clinical correlations, biological pathways, and variations in immune response among these risk categories. We also constructed a nomogram integrating clinical information with risk assessment. Focusing on the TENM1 gene, we found it significantly impacts immune response, showing a positive correlation with T helper cells, NK cells, and CD8+ T cells, but a negative correlation with neutrophils and Th17 cells. Gene Set Enrichment Analysis revealed enhanced pathways related to pancreatic beta cells, spermatogenesis, apical junctions, and muscle formation in patients with high TENM1 expression. This research provides new insights into the role of ECM genes in esophageal cancer and informs future research directions.

## 1. Introduction

Esophageal cancer (EC) is the sixth most common malignancy globally, characterized by a poor prognosis and high invasiveness [1]. Over 95% of EC cases are either squamous cell carcinomas or adenocarcinomas. Squamous cell carcinoma is more prevalent in developing countries, whereas adenocarcinoma is more common in developed countries [2]. The early symptoms of EC are often not apparent, leading to significant treatment delays [3]. A routine pathological biopsy performed under an endoscope is the most common diagnostic method. Although some patients may benefit from early surgery, recurrences and distant metastases can occur during subsequent adjuvant therapy [4]. In-depth studies of the tumor microenvironment can enhance the understanding of tumor genesis and progression, facilitating the discovery of therapeutic targets [5].

Tumor cells reside in a complex microenvironment known as the tumor microenvironment (TME). The extracellular matrix (ECM), a fundamental component of the TME, consists of various proteins secreted by cells, providing structural support and mediating cell interactions [6]. The abnormal ECM in the TME can affect the biological behaviors of cancer cells in multiple ways. According to a comprehensive review by Gilkes et al., changes in ECM content can directly influence its biological properties, contributing to cancer metastasis by affecting tumor cell heterogeneity [7]. Chaki et al. found that the interaction of Nck adapter proteins with downstream kinase 1 facilitates ECM degradation and cancer progression [8]. DiGiacomo et al. used a fibroblast-derived ECM scaffold for cell culture and discovered that the ECM scaffold significantly decreases the sensitivity of ER + breast cancer cells to ER-targeted therapy, a condition that can be reversed by the binding of FGF2 to FGFR1 [9]. The ECM is also regulated by immune cells. Haj-Shomaly et al. revealed that CD8+ T cells can induce ECM remodeling and cancer metastasis in paclitaxel-treated mice [10]. Tian et al. demonstrated that the microsome proteins derived from cancer cells AGRN, SERPINB5, and CSTB can promote pancreatic cancer metastasis and are associated with poor prognosis [11]. Additionally, the ECM and other cells in the TME can create a robust barrier around cancer cells in solid tumors, reducing the effectiveness of immunotherapy [12]. Drugs targeting the ECM can disrupt collagen fiber arrangement, enhancing immune cell infiltration and the efficacy of therapeutic drugs. [13].

A wealth of publicly available high-throughput datasets facilitates secondary data analyses and research. In this study, we investigated the role of ECM-related genes in EC. Using various algorithms and analyses, we identified several ECM-related genes—IBSP, LINGO4, COL26A1, MMP12, KLK4, RTBDN, TENM1, GDF15, and RUNX1—as significantly associated with patient survival. We developed a prognostic signature that effectively differentiates between high- and low-risk patient groups in terms of survival outcomes. Detailed analyses, including clinical correlation, biological enrichment, and immune infiltration, were performed to delineate the distinctions between these groups. Additionally, we combined clinical data and risk scores to construct a nomogram that exhibited superior predictive performance. Notably, TENM1 was selected for further investigation. Immunohistochemistry results revealed that TENM1 protein levels were reduced in EC tumor tissues. Moreover, immune infiltration analysis demonstrated a positive correlation of TENM1 with T helper cells, NK cells, and CD8+ T cells and a negative correlation with neutrophils and Th17 cells. Gene Set Enrichment Analysis (GSEA) showed that pathways related to pancreas beta cells, spermatogenesis, apical surface, and myogenesis were upregulated in patients with high levels of TENM1.

## 2. Methods

### 2.1. Open-Accessed Data Collection

Genomic and clinical data for EC patients were sourced from The Cancer Genome Atlas (TCGA) program, specifically the TCGA-ESCA project. Individual expression profiles (STAR-COUNTS) and clinical data were accessed via the TCGA-GDC program. For accurate probe annotation, the latest human genomic annotation file (GRCh38.p13) was downloaded from the ENSEMBL database. We excluded genes with a median expression value below 0.1 to ensure robust data quality. To address the skewness in gene expression data, we transformed the expression matrix using the log2 scale after adding a pseudo-count of 1. Mutation data for the genome were also retrieved from the TCGA database. Data preprocessing and analysis of differentially expressed genes were conducted using the Limma package, following specified thresholds. The tumor stemness index, mRNAsi, was obtained from the supplementary information of a prior study [14]. Due to the limited availability of normal tissue samples in the TCGA database, additional normal tissue data from the GTEx database were included in the analysis. The baseline characteristics of the enrolled patients are presented in Table 1.

### 2.2. Protein Interaction Network

Potential interactions among coding proteins were explored using the STRING database, with search parameters specifically set for “Homo sapiens” to ensure species-specific relevance [15].

### 2.3. Biological Enrichment Analysis

Biological enrichment analysis was performed using the ClueGO plugin within the Cytoscape software, focusing on significantly enriched terms (*P* < 0.01) to facilitate effective visualization and interpretation [16]. Gene ontology (GO) analysis was conducted using the R package clusterProfiler in the RStudio environment, applying filter criteria of “*P* value <0.05” and “*q* value <0.05” [17]. Furthermore, GSEA was employed to delineate biological differences between two groups, referencing specific pathway sets [18].

### 2.4. Clinical and Prognosis Analysis

Prognostic factors were initially identified using univariate Cox regression analysis with a significance level set at *P* < 0.05. The identified genes were further refined using LASSO regression analysis to optimize the selection of prognostic variables [19]. Subsequently, a multivariate Cox regression model was developed to ascertain independent prognostic factors. We constructed a predictive model using the formula: “Risk score = ∑(Coeff_*i* × Expression_*i*) for each significant gene *i*,” thereby enabling precise risk stratification. We also examined clinical correlations with patient characteristics such as gender, clinical stage, and TNM classification. Kaplan-Meier survival curves and receiver operating characteristic (ROC) curves were utilized to evaluate the prognostic accuracy of the model.

### 2.5. Establishment of Nomogram

A nomogram was developed to quantitatively predict patient survival using the rms package in RStudio. The predictive performance of the nomogram was evaluated through calibration curves, comparing predicted survival probabilities with observed outcomes.

### 2.6. Exploration of TME

The relative abundance of immune and stromal cells in the TME of EC patients was analyzed using the estimate package in R. Additionally, various algorithms including CIBERSORT, XCELL, EPIC, MCPCOUNTER, QUANTISEQ, TIMER, and ssGSEA algorithm were employed to evaluate immune cell infiltration levels in the EC TME [20].

### 2.7. Specific Drug Sensitivity

Sensitivity to immunotherapy in EC patients was determined using the Tumor Immune Dysfunction and Exclusion (TIDE) algorithm [21]. Sensitivity to targeted drugs was assessed using data from the Genomics of Drug Sensitivity in Cancer database. [22].

### 2.8. Single-Cell Analysis

Single-cell RNA sequencing data analysis was performed to explore the cellular heterogeneity and the specific expression patterns of TENM1 in EC. We utilized the TISCH database, a comprehensive resource for tumor-infiltrating single-cell transcriptomics. This platform allowed us to conduct an online analysis to identify the specific cell types expressing TENM1 within the tumor microenvironment of EC patients.

### 2.9. Cell Culture and Maintenance

EC cell lines (EC9706, KYSE150, YES2) and normal esophageal epithelial cells (HET-1A) were cultured in corresponding medium supplemented with 10% fetal bovine serum, 100 U/ml penicillin, and 100 *μ*g/ml streptomycin. Cells were maintained in a humidified incubator at 37°C with 5% CO2. The medium was changed every two days, and cells were passaged upon reaching 80–90% confluence using 0.25% trypsin-EDTA for detachment.

### 2.10. TENM1 Expression Analysis in EC Cells

To assess the mRNA and protein expression levels of TENM1 in EC cells, we conducted quantitative real-time PCR (qRT-PCR) and Western blot analysis. For mRNA analysis, total RNA was extracted from both EC cell lines (EC9706, KYSE150, and YES2) and normal esophageal epithelial cells (HET-1A), using the RNeasy Mini Kit (Qiagen). cDNA was synthesized using the High Capacity cDNA Reverse Transcription Kit (Applied Biosystems). qRT-PCR was performed using SYBR Green Master Mix (Thermo Fisher Scientific) on a StepOnePlus Real-Time PCR System (Applied Biosystems), with GAPDH serving as the internal control. For protein expression analysis, cells were lysed in RIPA buffer containing protease inhibitors. Protein concentrations were determined using the BCA Protein Assay Kit. Equal amounts of protein were loaded onto SDS-PAGE gels, transferred to PVDF membranes, and probed with antibodies specific to TENM1 (proteintech, 21696-1-AP, 1 : 1500) and GAPDH (proteintech, 60004-1-Ig, 1 : 10000).

### 2.11. Statistical Analysis

All statistical analyses were performed using R. The Student's *t*-test was applied to normally distribute continuous variables, while the Mann–Whitney *U* test was used for those with non-normal distributions.

## 3. Results

### 3.1. The Expression Pattern of ECM-Related Genes in EC and Their Biological Role

The overall workflow of this study is presented in Figure S1. To account for the differences between cancerous and normal tissues, we initially investigated the expression patterns of ECM-related genes in EC. Our findings revealed that 91 ECM genes were downregulated, while 109 genes were upregulated in EC tumor tissue. GO analysis indicated that these ECM-related genes are involved in processes such as glycosaminoglycan binding (GO: 0005539), endopeptidase activity (GO: 0004175), extracellular structure organization (GO: 0043062), collagen catabolic process (GO: 0030574), cell-substrate adhesion (GO: 0031589), collagen metabolic process (GO: 0032963), basement membrane (GO: 0005604), collagen trimer (GO: 0005581), Golgi lumen (GO: 0005796), extracellular matrix organization (GO: 0030198), endoplasmic reticulum lumen (GO: 0005788), laminin complex (GO: 0043256), and extracellular matrix disassembly (GO: 0022617) (Figure S2A). ClueGO analysis further demonstrated that these ECM-related genes were predominantly enriched in organ growth, chondrocyte differentiation, glycosaminoglycan catabolic process, skeletal system development, regulation of cell adhesion, cell-substrate adhesion, and extracellular matrix organization (Figure S2B).

### 3.2. Identification of the ECM-Related Genes Remarkably Affecting Patients' Survival

**Figure 1:**
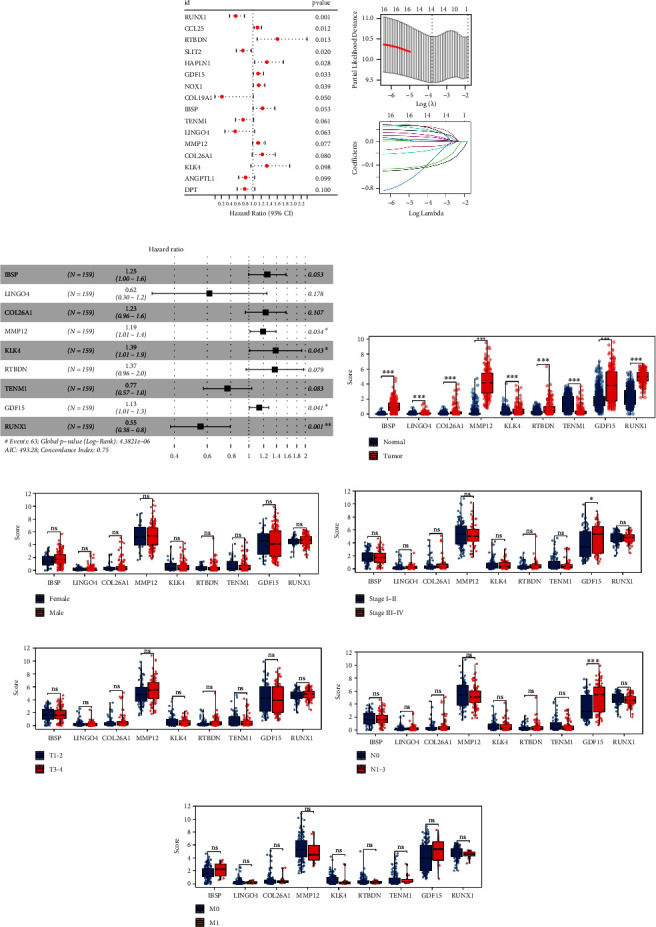
Identification of the ECM-related genes associated with patients survival. *Notes*. (a) Univariate Cox regression analysis was utilized to identify the prognosis-related genes with *P* < 0.05; (b) LASSO regression analysis; (c) multivariate cox regression analysis based on the genes screened by LASSO regression; (d–i) clinical correlation of IBSP, LINGO4, COL26A1, MMP12, KLK4, RTBDN, TENM1, GDF15, and RUNX1.

First, we conducted univariate Cox regression analysis with a significance threshold of *P* < 0.1. Significant impacts on patient survival were observed for the genes RUNX1, CCL25, RTBDN, SLIT2, HAPLN1, GDF15, NOX1, COL19A1, IBSP, TENM1, LINGO4, MMP12, COL26A1, KLK4, ANGPTL1, and DPT (Figure 1(a)). For further data dimension reduction, LASSO logistic regression was employed (Figure 1(b)). Subsequently, the genes IBSP, LINGO4, COL26A1, MMP12, KLK4, RTBDN, TENM1, GDF15, and RUNX1 were selected for inclusion in the prognostic model (Figure 1(c)). We then assessed the clinical correlation of these signature genes. The findings revealed that all selected genes except TENM1, which was downregulated, were overexpressed in EC tumor tissues (Figure 1(d)). No significant differences in gene expression were observed between female and male patients (Figure 1(e)). Higher levels of GDF15 were noted in patients at stages III-IV (Figure 1(f)) and in those with N1-3 EC (Figure 1(h)), whereas no significant expression differences were identified between T1-2 and T3-4 or between M0 and M1 patients (Figures 1(g), 1(h), and 1(i)).

### 3.3. Evaluation of Prognosis Model

**Figure 2:**
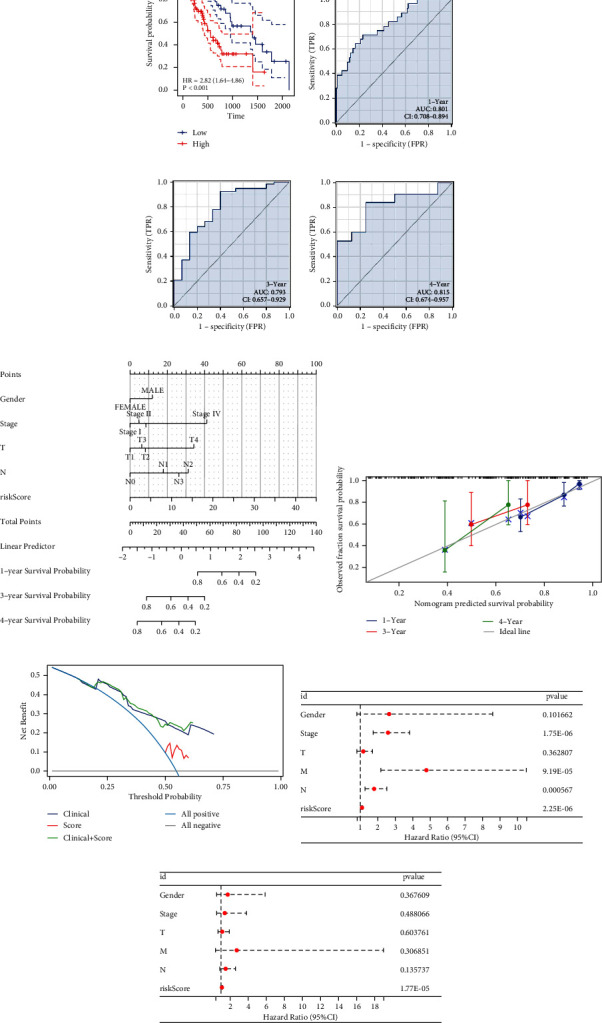
Performance of identified prognosis signature. *Notes*. (a) KM survival curves of patients in high- and low-risk groups; (b–d) ROC curve of 1-, 3-, and 5-year survival; (e) the nomogram plot combining risk score and clinical features; (f) calibration plot; (g) DCA curve; (h) univariate analysis; (i) multivariate analysis.

The risk score was computed using the equation: “Risk score = IBSP *∗* 0.226 + LINGO4 *∗* −0.485 + COL26A1 *∗* 0.204 + MMP12 *∗* 0.173 + KLK4 *∗* 0.328 + RTBDN *∗* 0.318 + TENM1 *∗* −0.261 + GDF15 *∗* 0.124 + RUNX1 *∗* −0.595.” KM curves revealed that patients with high-risk scores exhibited poorer survival outcomes (Figure 2(a), HR = 2.82). ROC curve analysis confirmed the robust predictive capability of our signature for patient survival, with AUC values of 0.801 at 1 year, 0.793 at 3 years, and 0.815 at 4 years (Figures 2(b), 2(c), and 2(d)). Subsequently, integrating clinical features with the risk score, a nomogram was constructed (Figure 2(e)). The calibration curve showed a strong agreement between actual survival and the predictions of the nomogram (Figure 2(f)). DCA demonstrated that the nomogram significantly enhances the predictive accuracy of the risk score (Figure 2(g)). Both univariate and multivariate analyses established that the risk score is an independent predictor of patient survival (Figures 2(h) and 2(i)).

### 3.4. The Genomic Difference in Different EC Patients

**Figure 3:**
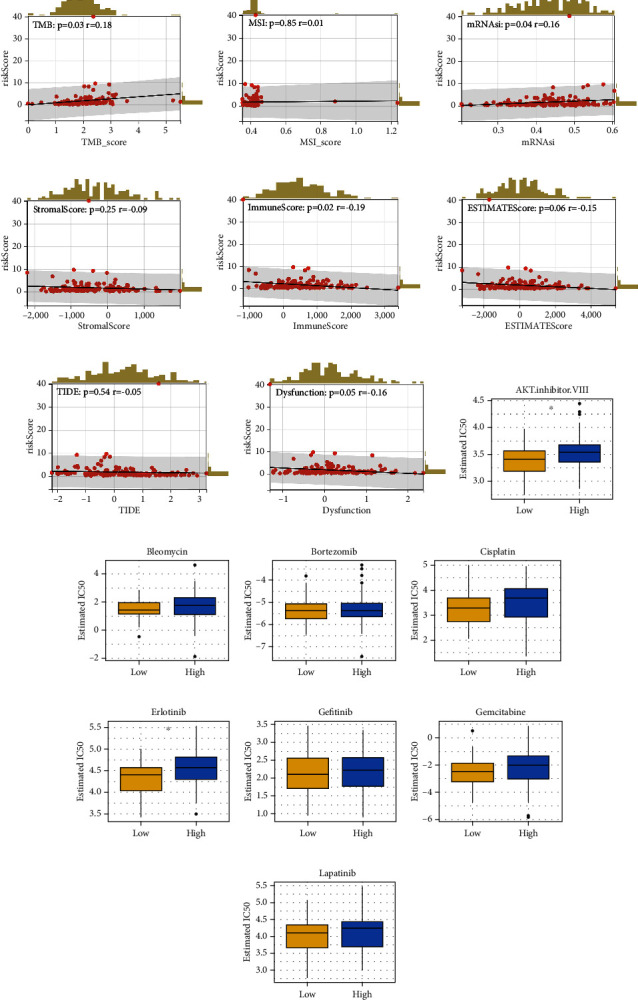
Immunotherapy and drug sensitivity analysis. *Notes*. (a–c) Correlation of risk score and TMB, MSI, and mRNAsi; (d–f) correlation of risk score and immune score, stromal score, and estimate score; (g) correlation between TIDE score and risk score; (h) correlation between risk score and immune dysfunction; (i–p) drug sensitivity analysis between high- and low-risk groups.

Genomic differences can lead to varied cell behaviors. Consequently, we aimed to elucidate the prognostic variations from a genomic standpoint. A positive correlation between the risk score and tumor mutational burden (TMB) was observed, suggesting that patients with high-risk scores might exhibit progressive genomic mutations (Figure 3(a)). However, no significant correlations were found between microsatellite instability (MSI) and mRNAsi (Figures 3(b) and 3(c)). Additionally, while a negative correlation was evident between the risk score and immune score, such correlations were absent between the stromal score and ESTIMATE score (Figures 3(d), 3(e) and 3(f)).

### 3.5. Immunotherapy and Drug Sensitivity

We then sought to explore the differences in immunotherapy response and drug sensitivity among patient groups. However, the expression of key immune checkpoints showed no significant differences between high- and low-risk patients (Figure S3). Additionally, there was no statistically significant correlation between the risk score and the TIDE score, suggesting that the risk score does not significantly influence EC immunotherapy outcomes (Figure 3(g)). Interestingly, a slight correlation was observed between immune dysfunction and the risk score (Figure 3(h)). Drug sensitivity analysis revealed that patients in the low-risk group may be more responsive to AKT inhibitors and erlotinib (Figures 3(i), 3(j), 3(k), 3(l), 3(m), 3(n), 3(o), and 3(p)).

### 3.6. Biological Enrichment and Immune Microenvironment Analysis

**Figure 4:**
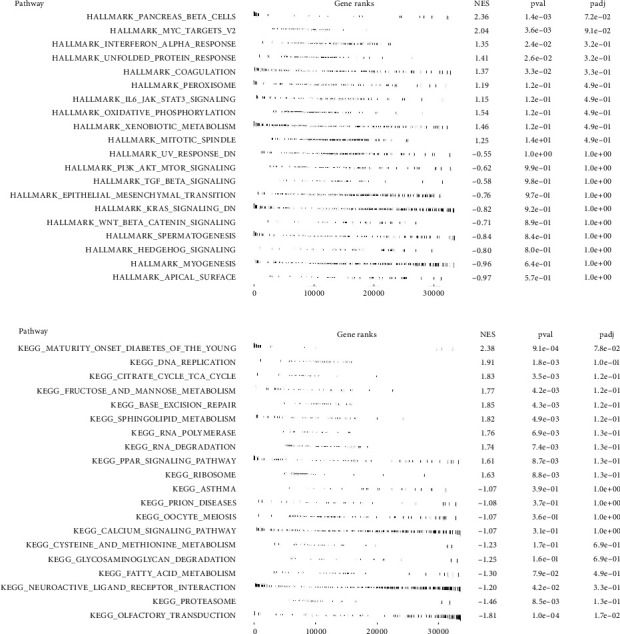
Biological enrichment analysis. *Notes*. (a) GSEA analysis based on hallmark gene set; (b) GSEA analysis based on KEGG gene set.

**Figure 5:**
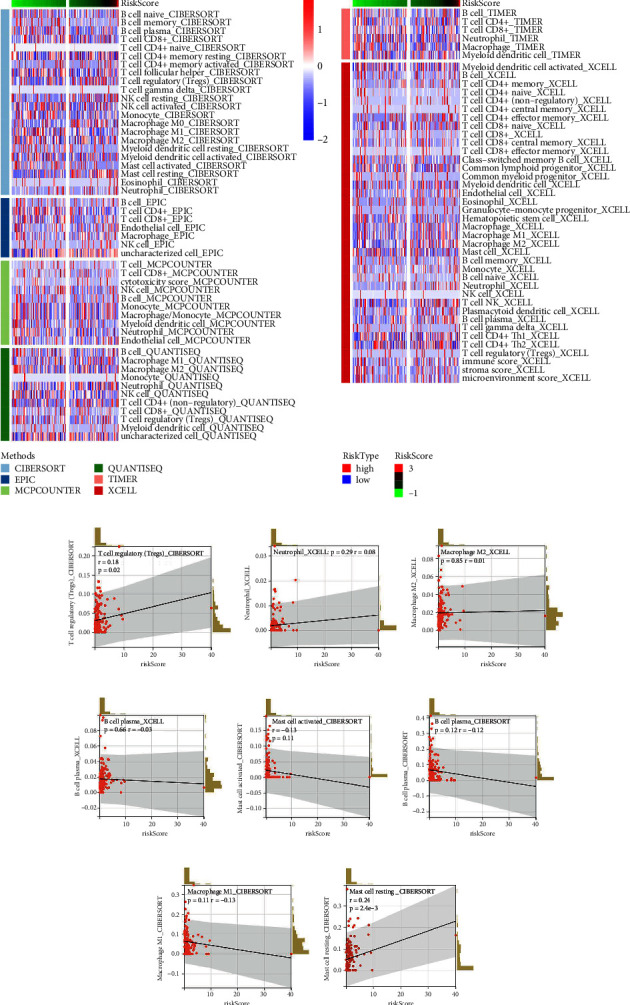
Immune infiltration analysis. *Notes*. (a) Quantification of immune cell infiltration based on multiple algorithms, including EPIC, CIBERSORT, MCPCOUNTER, QUANTISEQ, TIMER, and XCELL; (b–i): correlation of risk score and specific immune cells.

The progression and malignant behavior of cancer are influenced by various pathways and cascade reactions. Biological enrichment studies showed that pathways related to pancreas beta cells, coagulation, peroxisomes, IL6/JAK/STAT3 signaling, and oxidative phosphorylation were activated in high-risk patients (Figure 4(a), Hallmark). GSEA based on the Kyoto Encyclopedia of Genes and Genomes (KEGG) revealed that pathways associated with maturity-onset diabetes of the young, DNA replication, the citrate (TCA) cycle, base excision repair, and sphingolipid metabolism were enriched in these patients (Figure 4(b), KEGG). Leveraging multiple algorithms, we mapped the immune infiltration landscape of EC patients (Figure 5(a)). Analysis of correlations showed that the risk score positively correlated with Tregs, neutrophils, and resting mast cells but negatively correlated with activated mast cells, plasma B cells, and M1 macrophages (Figures 5(b), 5(c), 5(d), 5(e), 5(f), 5(g), 5(h), and 5(i)).

### 3.7. Further Exploration of TENM1 in EC

**Figure 6:**
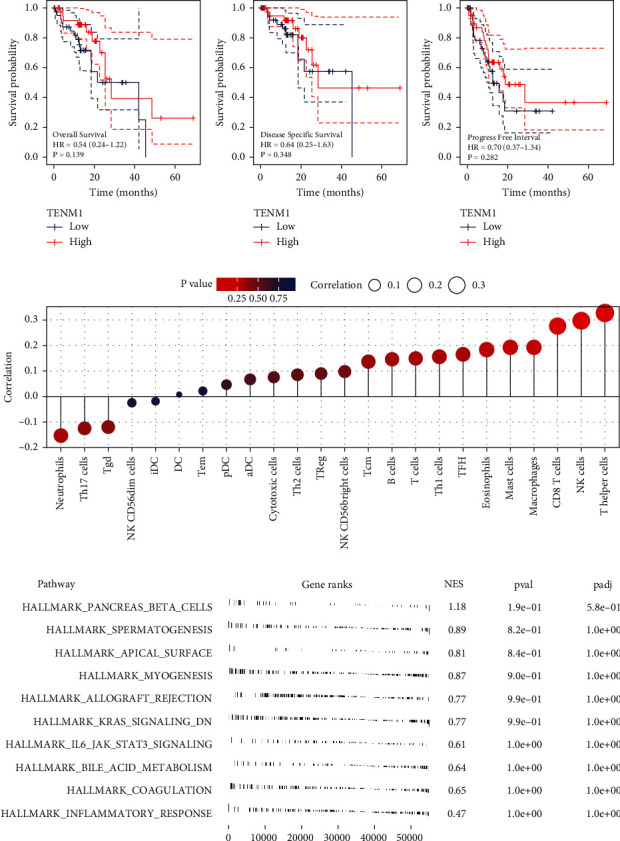
Further exploration of TNEM1 in EC. *Notes*. (a) KM curve of overall survival in patients with high and low TNEM1 expression; (b) KM curve of disease-free survival in patients with high- and low-TNEM1 expression; (c) KM curve of progression-free survival in patients with high- and low-TNEM1 expression; (d) the ssGSEA algorithm was utilized to illustrate the immune infiltration differences in patients with high- and low-TNEM1 expression; (e) GSEA analysis of TNEM1.

TENM1 has not been previously reported in the literature. Consequently, TENM1 was selected for further analysis in EC. Prognostic analysis revealed that TENM1 had no significant impact on overall survival, disease-free survival, or progression-free survival in patients (Figures 6(a), 6(b), 6(c)). However, the number of samples may affect these outcomes; thus, these results should be interpreted with caution. ssGSEA demonstrated a positive correlation between TENM1 and T helper cells, NK cells, and CD8+ T cells, while it showed a negative correlation with neutrophils and Th17 cells (Figure 6(d)). GSEA revealed that pathways related to pancreas beta cells, spermatogenesis, apical surface, and myogenesis were upregulated in patients with elevated TENM1 levels (Figure 6(e)). Single-cell analysis showed that TENM1 was mainly expressed in malignant and fibroblasts in EC microenvironment (Figures S4A–S4D).

### 3.8. Expression Level of TENM1 in EC Cells

**Figure 7:**
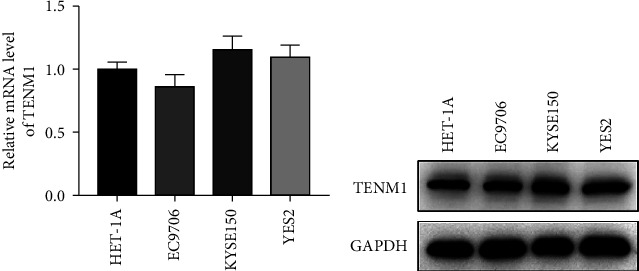
Expression level of TENM1 in EC cells. *Notes*. (a) The mRNA level of TENM1 in EC and normal cells; (b) the protein level of TENM1 in EC and normal cells.

Furthermore, we tried to detect the mRNA and protein expression level of TENM1 in EC cells. We found that there was no significant difference between EC cells and normal cells (HET-1A vs. EC9706, KYSE150, YES2) (Figures 7(a) and 7(b)).

## 4. Discussion

EC remains a significant global health threat [23]. For early-stage disease, surgical resection is the preferred treatment option. Nonetheless, there is still a high risk of postoperative recurrences and metastases [24]. In cases of advanced stages or recurrence, chemotherapy is commonly employed, although its benefits are somewhat limited [24]. Additionally, the adverse effects of chemotherapeutic drugs can partly hinder the successful treatment of EC. In the current biological era, advancements have facilitated disease understanding and the identification of novel therapeutic targets. Therefore, the identification of biomarkers that can guide the diagnosis and treatment of EC is crucial.

In this study, we explored the roles of ECM-related genes in EC. Using a series of algorithms and analyses, we identified several ECM-related genes—IBSP, LINGO4, COL26A1, MMP12, KLK4, RTBDN, TENM1, GDF15, and RUNX1—that are significantly associated with patient survival. We established a prognostic prediction signature that differentiates between high- and low-risk groups, reflecting varied survival outcomes. To elucidate the differences between these groups, we performed clinical correlation, biological enrichment, and immune infiltration analyses. Furthermore, we integrated clinical data with risk scores to develop a nomogram that demonstrates enhanced predictive accuracy. Notably, the gene TENM1 was selected for in-depth analysis. Immunohistochemistry revealed that TENM1 protein levels were downregulated in EC tumor tissues. Immune infiltration analysis indicated positive correlations of TENM1 with T helper cells, NK cells, and CD8+ T cells, and negative correlations with neutrophils and Th17 cells. GSEA showed that pathways related to pancreas beta cells, spermatogenesis, apical surface, and myogenesis were upregulated in patients with elevated TENM1 levels.

Our study identified the ECM-related genes IBSP, LINGO4, COL26A1, MMP12, KLK4, RTBDN, TENM1, GDF15, and RUNX1 as significantly associated with patient survival. Several of these genes have been implicated in various cancers. For instance, in breast cancer, Wu et al. reported that IBSP, secreted from ER + breast cancer cells, fosters an osteoclast-rich microenvironment that supports the exocrine transport of miR-19a and enhances bone metastasis [25]. Lin et al. demonstrated that inhibition of MTA2 suppresses MMP12 expression via the ASK1/MEK3/p38/YB1 pathway, thereby reducing the metastatic potential of cervical cancer cells [26]. In EC, Hu et al. showed that KLK4 regulation by RP11-465B22.8 through miR-765 contributes to cancer progression [27]. Similarly, Sun et al. found that miR-486 curtails the progression of papillary thyroid carcinoma by downregulating TENM1 and influencing the ERK and AKT pathways [28]. Dong et al. reported that GDF15 enhances the invasiveness of EC, mediated by SCAP [29]. Additionally, Wu et al. observed that the lncRNA uc002yug.2 facilitates the alternative splicing of RUNX1, impacting EC progression [30]. Not all model genes have been studied in EC; our findings may guide further research in this area.

Our results indicated that in high-risk patients, pathways including those of pancreatic beta cells, MYC targets, interferon alpha response, unfolded protein response, coagulation, peroxisome, IL6/JAK/STAT3 signaling, and oxidative phosphorylation were upregulated. Ma et al. observed that ANXA2 promotes EC development by activating the MYC/HIF1A/VEGF axis [31]. Additionally, Li et al. reported that cloperastine inhibits EC proliferation by modulating oxidative phosphorylation [32]. Gong et al. described that circPUM1, originating from the nuclear genome, regulates oxidative phosphorylation and impacts EC cell death [33]. We also observed increased genomic instability in high-risk patients, a well-known cancer hallmark. This heightened instability often leads to more aggressive cancer behavior. Correlation analyses showed that risk scores were positively associated with Tregs and resting mast cells. Generally, Tregs contribute to creating an inhibitory immune microenvironment. Wang et al. demonstrated that CCL20, secreted by colon cancer cells, enhances chemotherapy resistance by promoting Treg infiltration [34]. Similarly, Li et al. discovered that a specific formula reduces breast cancer metastasis by inhibiting Treg differentiation and infiltration, which is induced by TAM/CXCL1 [35].

Drug sensitivity analysis revealed that patients classified as low-risk exhibited greater sensitivity to AKT inhibitors VIII and erlotinib compared to their high-risk counterparts. These low-risk patients likely possess more stable genomic characteristics, which may influence drug sensitivity, although the underlying mechanisms remain unclear. Prior research suggests that genomic features can impact erlotinib's efficacy; for example, Lu et al. identified mutations in lung cancer that modulate the drug's response [36]. Similarly, Cai et al. reported that genetic alterations in breast cancer could reduce sensitivity to PI3K*α* inhibitors [37]. Additionally, we observed differences in specific pathways between high and low-risk patients, some of which have been previously associated with erlotinib response. For instance, Karaca et al. demonstrated a link between the Wnt/*β*-catenin signaling pathway and erlotinib's promotive effects in endometrial cancer cells [38]. These biological variances likely contribute to the heightened sensitivity of low-risk patients to AKT inhibitors VIII and erlotinib.

Despite the rigorous nature of our analysis, this study has several limitations. First, our research sample predominantly consists of individuals from Western populations, which may introduce racial bias and limit the generalizability of our findings. Second, the presence of incomplete clinical data may lead to inherent biases, although we anticipate that more comprehensive clinical characteristic data in the future will enhance the reliability of our findings. Third, our validation was limited to the protein level of TNEM1 in EC. Future studies should explore additional molecules to broaden our understanding.

## Figures and Tables

**Table 1:** Baseline information of enrolled patients.

Characteristic		Number	Percentage (%)

Gender	Female	27	14.8
	Male	156	85.2

Stage	Stage I	18	9.8
	Stage II	78	42.6
	Stage III	55	30.1
	Stage IV	9	4.9
	Unknown	23	12.6

Tstage	T0	1	0.5
	T1	31	16.9
	T2	43	23.5
	T3	86	46.9
	T4	5	27.8
	Unknown	17	9.3

Mstage	M0	134	73.2
	M1	9	4.9
	Unknown	40	21.9

Nstage	N0	76	41.5
	N1	68	37.2
	N2	12	6.6
	N3	8	4.4
	Unknown	19	10.4

## Data Availability

The data used to support the findings of this study are available from the corresponding authors upon reasonable request.

## Supplementary Materials

Supplementary Materials Figure S1 shows the flowchart of whole study. Figure S2 shows the expression pattern and biological role of ECM-related genes. Figure S3 shows the immune checkpoint difference between high- and low-risk groups. Figure S4 shows the single-cell level of TENM1 in EC microenvironment.
