# Supplementary material for: Impact of Extracellular Matrix-Related Genes on the Tumor Microenvironment and Prognostic Indicators in Esophageal Cancer: A Comprehensive Analytical Study
Source: Genet Res (Camb). 2024 Jul 25;2024:3577395. doi: 10.1155/2024/3577395 (PMC11300105; doi:10.1155/2024/3577395)

Supplementary Description

Figure S1. The flowchart of the whole study

Figure S2. The expression pattern and biological role of ECM-related genes

Notes: A: GO analysis of ECM-related genes; B: ClueGO analysis of ECM-related genes.

Figure S3. The immune checkpoint difference between high- and low-risk groups

Figure S4. The single-cell expression of TENM1 in EC microenvironment

Notes: A-B: Single-cell level of TENM1 in GSE16029; C-D: Single-cell level of TENM1 in GSE173590.

Figure S1. The flow chart of whole study

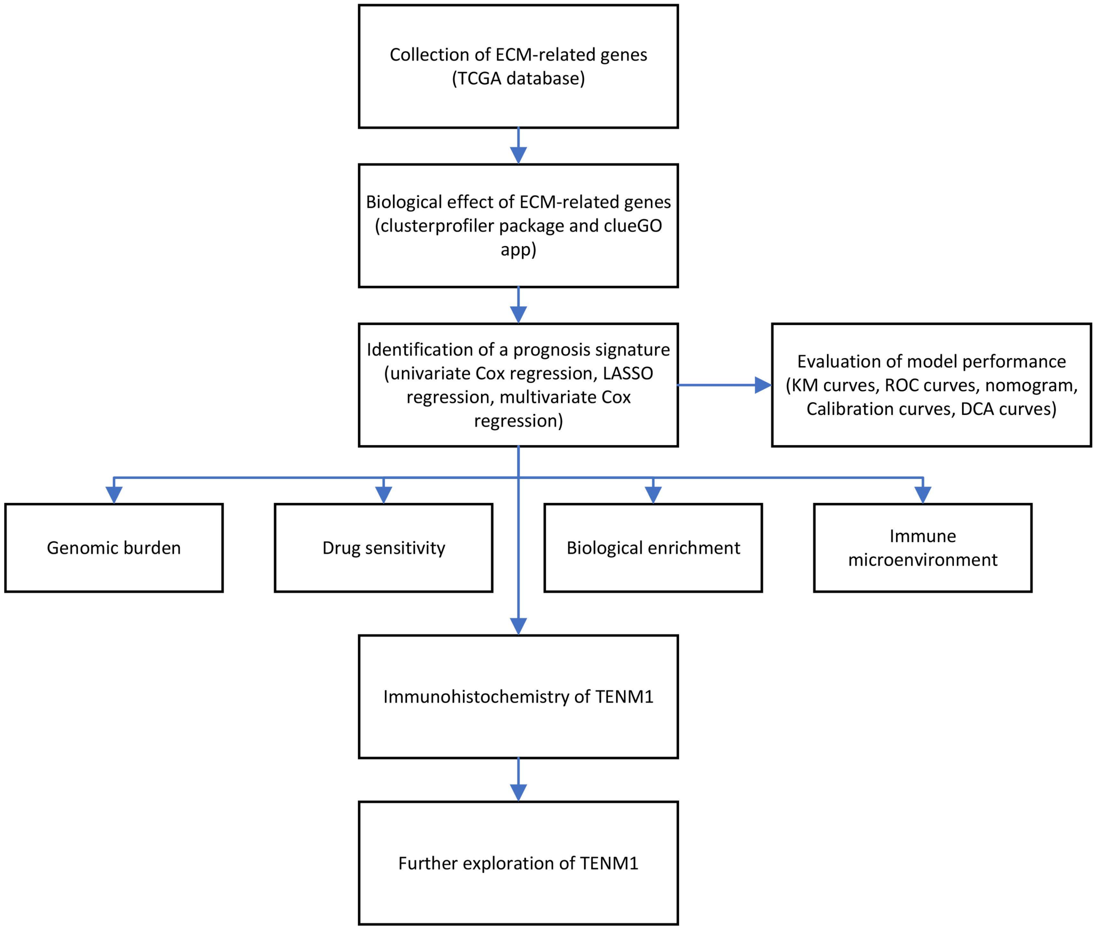

Supplement: Supplementary Materials — Figure S1 shows the flowchart of whole study. Figure S2 shows the expression pattern and biological role of ECM-related genes. Figure S3 shows the immune checkpoint difference between high- and low-risk groups. Figure S4 shows the single-cell level of TENM1 in EC microenvironment. [file 3577395.f1.zip › Figure S1.pdf]
