# Supplementary material for: Impact of Extracellular Matrix-Related Genes on the Tumor Microenvironment and Prognostic Indicators in Esophageal Cancer: A Comprehensive Analytical Study
Source: Genet Res (Camb). 2024 Jul 25;2024:3577395. doi: 10.1155/2024/3577395 (PMC11300105; doi:10.1155/2024/3577395)
Supplement: Supplementary Materials — Figure S1 shows the flowchart of whole study. Figure S2 shows the expression pattern and biological role of ECM-related genes. Figure S3 shows the immune checkpoint difference between high- and low-risk groups. Figure S4 shows the single-cell level of TENM1 in EC microenvironment. [file 3577395.f1.zip › Figure S4.pdf]

Figure S4

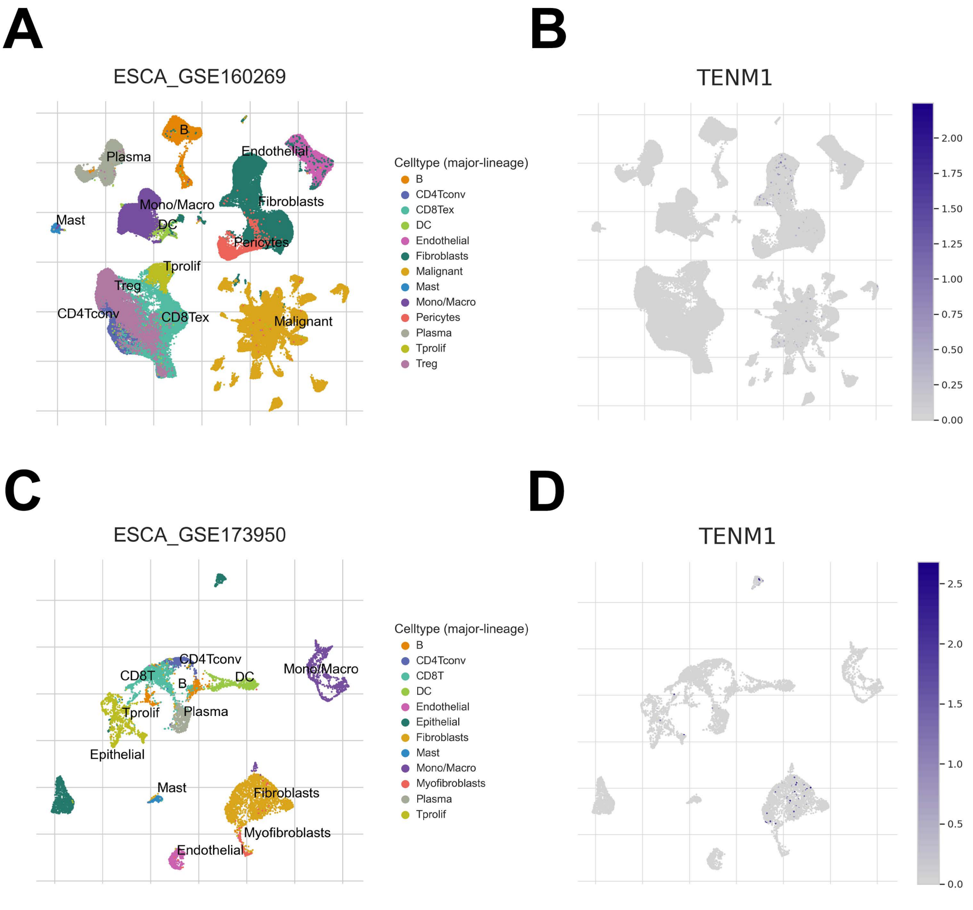

A
ESCA_GSE160269
B
Plasma
Endothelial
Mono/Macro
Fibroblasts
Mast
DC
Pericytes
Tprolif
Treg
CD4Tconv
CD8Tex
Malignant
Celltype (major-lineage)
B
CD4Tconv
CD8Tex
DC
Endothelial
Fibroblasts
Malignant
Mast
Mono/Macro
Pericytes
Plasma
Tprolif
Treg
B
TENM1
2.00
1.75
1.50
1.25
1.00
0.75
0.50
0.25
0.00
C
ESCA_GSE173950
CD4Tconv
CD8T
B
DC
Mono/Macro
Tprolif
Plasma
Epithelial
Mast
Fibroblasts
Myofibroblasts
Endothelial
Celltype (major-lineage)
B
CD4Tconv
CD8T
DC
Endothelial
Epithelial
Fibroblasts
Mast
Mono/Macro
Myofibroblasts
Plasma
Tprolif
D
TENM1
2.5
2.0
1.5
1.0
0.5
0.0
