# Supplementary figures and images for: Impact of Extracellular Matrix-Related Genes on the Tumor Microenvironment and Prognostic Indicators in Esophageal Cancer: A Comprehensive Analytical Study
Source: Genet Res (Camb). 2024 Jul 25;2024:3577395. doi: 10.1155/2024/3577395 (PMC11300105; doi:10.1155/2024/3577395)

Figure S2

A

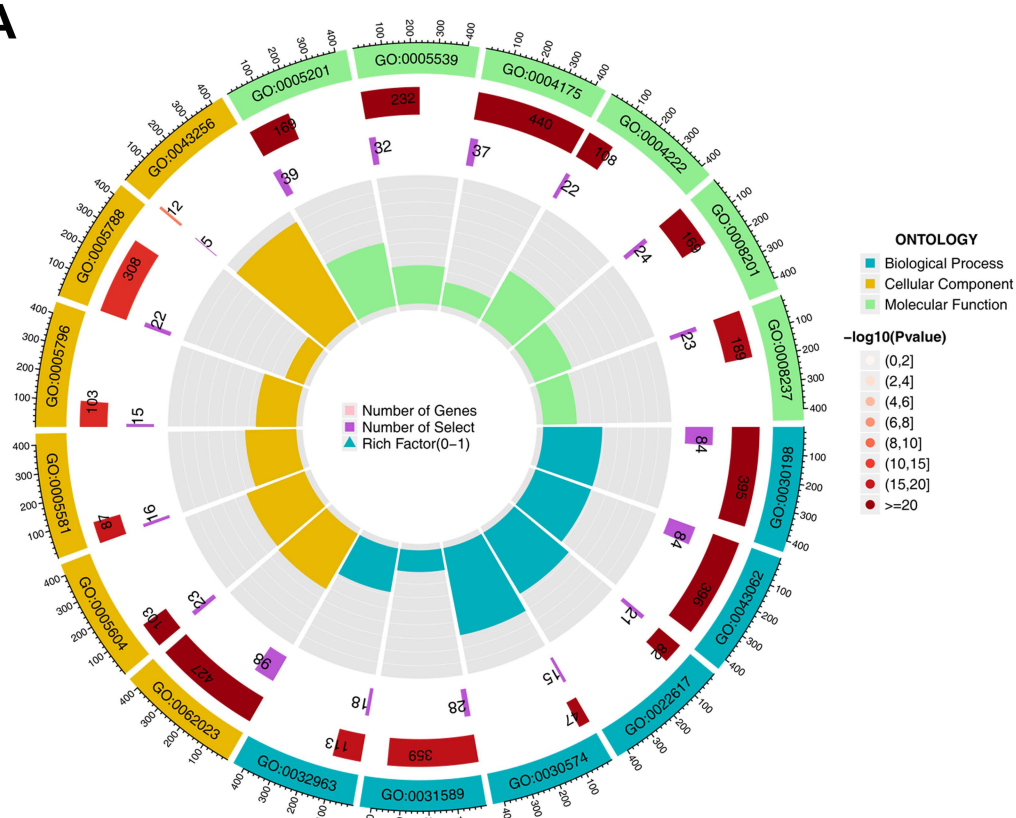

B

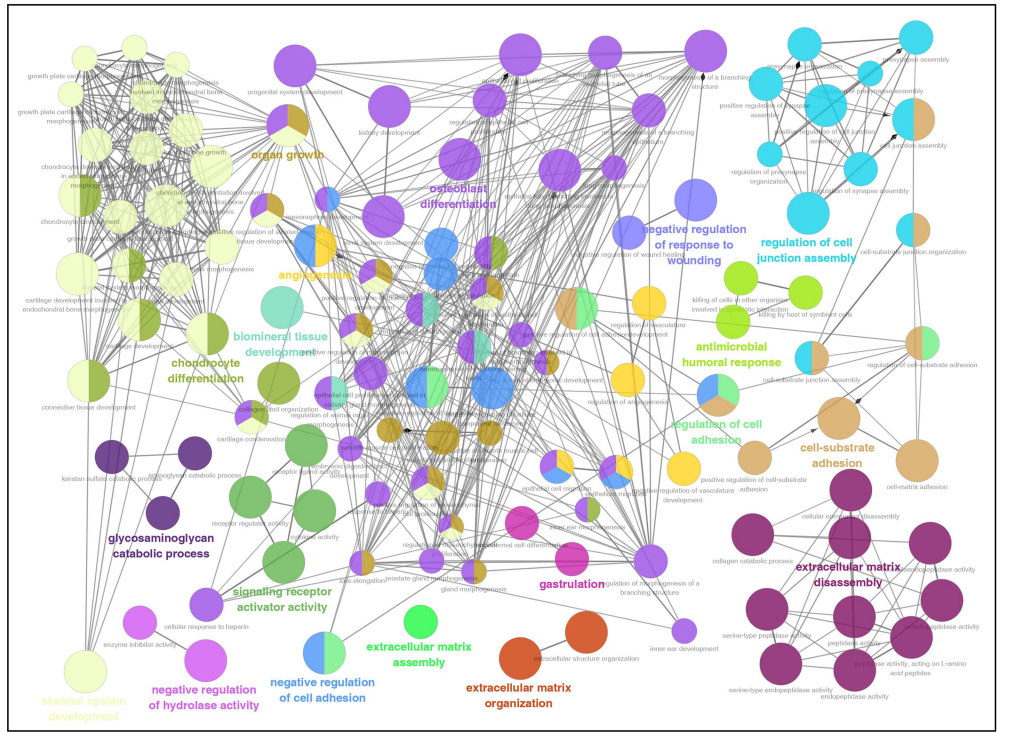

Supplement: Supplementary Materials — Figure S1 shows the flowchart of whole study. Figure S2 shows the expression pattern and biological role of ECM-related genes. Figure S3 shows the immune checkpoint difference between high- and low-risk groups. Figure S4 shows the single-cell level of TENM1 in EC microenvironment. [file 3577395.f1.zip › Figure S2.pdf]

Figure S3

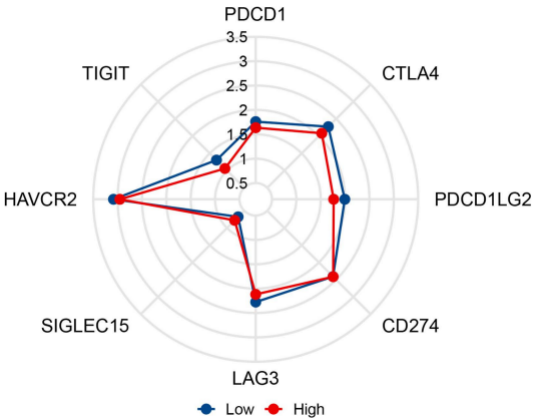

Supplement: Supplementary Materials — Figure S1 shows the flowchart of whole study. Figure S2 shows the expression pattern and biological role of ECM-related genes. Figure S3 shows the immune checkpoint difference between high- and low-risk groups. Figure S4 shows the single-cell level of TENM1 in EC microenvironment. [file 3577395.f1.zip › Figure S3.pdf]
